# The training and professional expectations of medical students in Angola, Guinea-Bissau and Mozambique

**DOI:** 10.1186/1478-4491-9-9

**Published:** 2011-04-07

**Authors:** Paulo Ferrinho, Mohsin Sidat, Mário Jorge Fresta, Amabélia Rodrigues, Inês Fronteira, Florinda da Silva, Hugo Mercer, Jorge Cabral, Gilles Dussault

**Affiliations:** 1 Associação para o Desenvolvimento e Cooperação Garcia de Orta, Lisbon, Portugal; 2 Health Systems Unit and Center for Malaria and Other Tropical Diseases, Instituto de Higiene e Medicina Tropical, Universidade Nova de Lisboa, Lisbon, Portugal; 3 Faculty of Medicine, University Eduardo Mondlane, Maputo, Mozambique; 4 Cedumed, Faculty of Medicine, University Agostinho Neto, Luanda, Angola; 5 National Institute of Public Health, Bissau, Guinea-Bissau; 6 Instituto de Salud Pública, Universidad de Buenos Aires, Buenos Aires, Argentina

## Abstract

**Background:**

The purpose of this paper is to describe and analyze the professional expectations of medical students during the 2007-2008 academic year at the public medical schools of Angola, Guinea-Bissau and Mozambique, and to identify their social and geographical origins, their professional expectations and difficulties relating to their education and professional future.

**Methods:**

Data were collected through a standardised questionnaire applied to all medical students registered during the 2007-2008 academic year.

**Results:**

Students decide to study medicine at an early age. Relatives and friends seem to have an especially important influence in encouraging, reinforcing and promoting the desire to be a doctor.

The degree of feminization of the student population differs among the different countries.

Although most medical students are from outside the capital cities, expectations of getting into medical school are already associated with migration from the periphery to the capital city, even before entering medical education.

Academic performance is poor. This seems to be related to difficulties in accessing materials, finances and insufficient high school preparation.

Medical students recognize the public sector demand but their expectations are to combine public sector practice with private work, in order to improve their earnings. Salary expectations of students vary between the three countries.

Approximately 75% want to train as hospital specialists and to follow a hospital-based career. A significant proportion is unsure about their future area of specialization, which for many students is equated with migration to study abroad.

**Conclusions:**

Medical education is an important national investment, but the returns obtained are not as efficient as expected. Investments in high-school preparation, tutoring, and infrastructure are likely to have a significant impact on the success rate of medical schools. Special attention should be given to the socialization of students and the role model status of their teachers.

In countries with scarce medical resources, the hospital orientation of students' expectations is understandable, although it should be associated with the development of skills to coordinate hospital work with the network of peripheral facilities. Developing a local postgraduate training capacity for doctors might be an important strategy to help retain medical doctors in the home country.

## Background

The Portuguese-speaking African countries became independent from Portugal after 1975. Until the mid-1990s, their political systems were one-party systems, which gradually changed to multi-party systems. Three of these countries (Angola, Mozambique and Guinea-Bissau) went through periods of civil war. The introduction of multi-party systems brought major economic restructuring processes, including moving from a centrally-planned economy to a market economy. A plethora of new laws and regulations have been passed since then, liberalizing activities that previously were under State control, including the health services.

Medical education has tried to keep up with the changes in the health care system. Mozambique and Angola have had a medical faculty since colonial times. Since independence, these have produced doctors to partially meet the needs of an exclusively public sector 'socialist health care system', free of charge at the point of delivery. Recently, efforts were made to adapt medical curricula to a new vision of a system where other social partners emerge as providers of health care and as trainers of medical students. In Mozambique, three new medical schools have been established outside Maputo: the private Catholic University established a medical college in Beira (central region), in 2001; the Universidade do Lúrio established a medical college in Nampula (northern region), in 2007; and, more recently, another public medical school has been established in Tete (eastern region). In 2009, in Angola, six new medical schools were established outside Luanda, where there is also a private medical school.

The Raul Diaz Arguellez Faculty of Medicine in Guinea-Bissau, is not currently integrated in any University, and offers a training programme supported by Cuban tutors. It was, at the time of the study, in its third year of training.

The purpose of this research is to describe and analyse the profile of medical students currently (2007) in the medical faculties of the Universities of Eduardo Mondlane (Mozambique), Agostinho Neto (Angola) and Raul Diaz Arguellez (Guinea-Bissau), to identify where they come from and their expectations and difficulties regarding their education and their professional career.

## Methods

A piloted, standardized questionnaire, with closed and open-ended questions, was distributed to all the registered medical students on a specific day, during an agreed lecture period, in 2007 or 2008. Some of the questions were context-specific and adapted to the reality of each country. All data were entered into an Access database and analysed using SPSS. Statistical analysis was mostly descriptive.

## Results

### Students' background

The median age of students varied between 22 years (Mozambique) and 26 (Angola). With the exception of Guinea-Bissau, most were females (Table 1). Most students were born and received their primary and secondary school education in the Province/Region of the Capital City, where the medical school is located (with the exception of Guinea-Bissau, where medical training is decentralized to several locations). The trend of migration to the capital city most marked in Guinea-Bissau and less so in Angola.

**Table 1:** Demographic characteristics and decision to take a degree in medicine: percentage and number (in brackets) except where indicated

		Angola	Guinea-Bissau	Mozambique
	Mean (sd)	27.7(7.6)	25.3(3.2)	22.8 (3.8)
Age	Median	26	25	22
	Mode	22	23	20 and 21

	Male	37.4 (189)	69.1 (56)	49.8 (241)
Sex	Female	62.6 (317)	30.9 (25)	50.2 (243)
	Total	100.0(508)	100.0 (81)	100.0 (484)

	In the country	98.2 (494)	97.5 (78)	98.3 (468)
Place of birth	In the Capital city province/region	48.9 (246)	51.3 (41)	56.2(190)
	Abroad	1.8 (9)	2.4 (2)	1.7 (8)
	Total	100.0 (503)	100.0 (80)	100.0 (476)

	In the country	97.6 (491)	97.6 (79)	98.4 (473)
Primary school	In the Capital city province/region	54.5 (275)	53.1 (43)	62.7 (212)
	Abroad	2.4 (12)	2.4 (2)	1.6 (8)
	Total	100.0 (503)	100.0 (81)	100.0 (481)

	In the country	97.6 (494)	97.6 (79)	98.5(477)
Secondary school	In the Capital city province/region	58.1 (294)	81.5 (66)	63.9 (216)
	Abroad	2.4 (12)	2.4 (2)	1.5 (5)
	Total	100.0 (506)	100.0 (81)	100.0 (482)

	Mean (sd)	15.8 (5.9)	16.1 (4.4)	14.9 (5.1)
Age at decision to take a medical degree	Median	16	15	15
	Mode	15	15	18

### The decision to study medicine

The median age of taking the decision to study medicine was 15 years (Guinea-Bissau and Mozambique) and 16 years (Angola) (Table 1).

The main reasons to choose medicine as a profession were "to contribute to the welfare of the public", "self-realization", "vocation", "family influence/pressure" and "social recognition"

### Academic performance

Between 5% (Guinea-Bissau) and 20% (Mozambique) of students were repeating one or another subject (students surveyed in Mozambique included participants in the seventh year of training, whereas in Guinea-Bissau the training had just reached its fourth year) (Table 2). In Mozambique the most frequent problem was the physiology course.

**Table 2:** Year of attendance and delayed disciplines: percentage and number (in brackets)

		Angola	Guinea-Bissau	Mozambique
	1^st^	14.0 (71)	22.5 (18)	25.0 (121)
	2^nd^	19.7 (100)	77.5 (62)	24.4 (118)
	3^rd^	21.3 (108)	-	13.0 (63)
Year of training	4^th^	13.2 (67)	-	18.4 (89)
	5^th^	19.1 (97)	-	7.7 (37)
	6^th^	12.8 (65)	-	5.4 (26)
	7^th^	-	-	6.2 (30)
	Total	100.0 (508)	100.0 (80)	100.0 (484)

Students with delayed disciplines		12.4 (63)	5.0 (4)	20 (95)

The main reasons for having failed were mostly related to "lack of personal effort", "lack of tutoring", "difficulty with the subject matter", "personal problems" and "lack of study materials".

### Main difficulties reported

The most frequent difficulties reported by students during the medical training were: "lack of books", and "financial needs". Other difficulties were "lack of adequate technology", "teachers not adequately prepared", "inadequate syllabus" and "insufficient knowledge from undergraduate schooling", reflecting the poor level of knowledge imparted by high school education [1].

### Satisfaction with the academic education received

The main factor of dissatisfaction was related to the poor quality of support systems (library, computers, laboratories) and the heavy load (and poor organization) of formal teaching hours.

### Expectations regarding their future profession and professional income

When asked in which sector they would like to practice medicine, most reported both the public and private sectors (from 55.6% in Guinea-Bissau to 77.4% in Mozambique). Those who expressed the desire to work exclusively in the public sector exclusively ranged from between 19.3% in Mozambique to 44.4% in Guinea-Bissau; and a minority desired to work in the private sector exclusively (from 0% in Guinea-Bissau to 3.4% in Mozambique) (Table 3).

**Table 3:** Perspectives about the professional future of medical students: percentage and number (in brackets)

		Angola	Guinea-Bissau	Mozambique
	Private	1.8 (9)	0	3.4 (16)
Sector where students would like to work	Public	26.4 (131)	44.4 (36)	19.3 (92)
	Both	71.8 (356)	55.6 (45)	77.4 (369)

	Hospital	70.7 (341)	88.6 (70)	74.8 (353)
Level of care where students would like to work	Community	29.0 (140)	8.9 (7)	23.7 (112)
	Both	0.2 (1)	1.3 (1)	1.5 (7)

	Country of training	79.3 (403)	90.7 (75)	80.2 (388)
	Other African countries	0.8 (4)	1.3 (1)	1.4 (7)
	Europe	1.6 (8)	-	4.1 (20)
Country where students would like to work	North America	0.2 (1)	-	0.8 (4)
	Others	0.8 (4)	6.6 (5)	0.4 (2)
	Any country	0.2 (1)	-	1.2 (6)
	Did not answer	17.1 (87)	-	11.8 (57)
	Total	100.0 (508)	100.0 (81)	100.0 (484)

Over 70% wanted to work at hospital level, 10% to 30% at community level and a small proportion at both.

Over 70% stated the intention of remaining in their country to work, but most expressed the willingness to go abroad to specialize or pursue additional studies (Table 4).

**Table 4:** Country of preference for future specialization: percentage and number (in brackets)

		Angola	Guinea-Bissau	Mozambique
	Country of residence	11.9 (55)	7.5 (6)	12.2 (59)
	Other African countries	5.5 (25)	2.5 (2)	8.6 (42)
	Europe	21.8 (100)	10.0 (8)	28.9 (140)
Preferred country for specialization	North America	8.1 (37)		7.0 (34)
	Latin America	51.8 (238)	77.5 (62)	30.5 (148)
	Asia	-	2.5 (2)	3.3 (16)
	Don't know/no answer	0.4 (2)	-	9.3 (45)
	Other	0.9 (4)	-	-
	Total	100.0 (459)	100.0 (80)	100.0 (484)

Surgical specialities were among the three favourite areas of specialist training in the three countries. The most popular medical specialities were gynaecology and obstetrics and paediatrics (Table 5).

**Table 5:** Areas of preference for future specialization: percentage and number (in brackets)

		Angola	Guinea-Bissau	Mozambique
	Surgery	14.4 (73)	17.2 (14)	24.1 (112)
	Paediatrics	10.4 (53)	18.5 (15)	9.4 (44)
	Gynaecology	12.0 (61)	17.3 (14)	8.2 (38)
Preferred area of specialization	Public Health	1.6 (8)	1.2 (1)	1.5 (7)
	Medicine	16.1 (82)	8.6 (7)	11.8 (55)
	Basic sciences	0.6 (3)	1.2 (1)	0.9 (4)
	Other	3.7 (19)	2.4 (2)	1.5 (7)
	Do not know	41.1 (209)	21.0 (17)	40.4 (188)
	Total	100.0 (508)	100.0 (71)	100.0 (455)

Responses on what they would consider a fair level of monthly income after graduation, are available only for Guinea-Bissau and Mozambique. As the income brackets used for each country were different, it is difficult to compare the responses. In Guinea-Bissau, where the starting monthly salary of a public sector doctor was US$ 320, 32% of future doctors expected to earn monthly up to US$ 416, and 8% expected to earn more than US$ 1667 per month in the first year after graduation. In Mozambique, where the starting salary of a public sector doctor was US$ 330 per month, only 8.6% of respondents expected to earn less than US$ 462 monthly, whereas 23.5% expected to earn more than US$ 1538 per month.

## Discussion

The urban migration documented during primary and secondary school education sets the scene for admission into medical school. It is indicative of the need to focus on primary and secondary school education to allow for the recruitment of medical students that received their education in environments where they will be most needed as doctors later on.

The feminization tendency observed among medical students in this study is described in a previous study in Mozambique [2] and also from other African Faculties of Medicine [3].

The degree of satisfaction remains, in Mozambique, similar to that reported in a recent study by Sousa et al. [2]. A significant percentage of students were repeating at least one subject, a problem also reported by other African medical faculties [4-9].

In Transkei, South Africa, in 2002, it was reported that at least 40% of students were not sure of their future area of specialization [3]. This study confirms the little interest shown by medical students in basic sciences [8].

Our results also correspond to Dambisya's findings [3] that most students would prefer to settle for hospital-based practice and work in the public sector.

About 10% to 20% of the students would like to emigrate to practice abroad, similar to the findings in Transkei, South Africa [3], but much lower than the emigration intentions of students from the Faculty of Medicine in Johannesburg, South Africa [10].

As far as income is concerned, most students would like to earn a salary much above the income offered by a public sector job, creating the context to encourage the overlap of public and private practice.

## Conclusions

The results from this study suggest that in countries with an acute shortage of medical graduates, and which invest a large share of their scarce resources into medical training, it might be wise to prioritize medical graduates for work in hospitals, whereas other categories should be deployed to primary health care facilities. Parallel attention to training in community health could prepare the doctors-to-be to enjoy periods of work at district hospitals, providing technical back-up to population-based interventions, which could be particularly beneficial in rural areas.

A chain of investments from primary school to college is necessary to obtain results in medical education (such as recruitment, socialization of students, material conditions, organization of academic life, and teachers as role models).

In many other African countries, the critical step in the migration of medical graduates is the moment when they decide to obtain specialised training: a frequent individual decision is to look for it abroad, leading to a subsequent decision to stay in the receiving country [11]. The results from this study also reflect a common picture: although only a small percentage of respondents express the wish to work abroad, a large majority would like to obtain specialized training outside their country of origin. It can therefore be suggested that investments to create capacity to undertake specialized training can become a useful tool to control the brain-drain.

The aforementioned suggestions may seem like a covert justification for the common practice of directing too much health expenditure towards hospitals. However, countries still facing an extreme shortage of medical graduates have the right to seek cost-effectiveness from the investment being made in medical education: the output of medical education - doctors is a scarce and expensive resource that must be retained in the country, and at the institutional level, where they are most relevant.

## Competing interests

The authors declare that they have no competing interests.

## Authors' contributions

PF was responsible for the all study and drafted the manuscript. MS, MJF and AR participated in the study design and data collection in Mozambique, Angola and Guinea-Bissau, respectively. IF performed data analysis. FS, HM supported field work. JC and GD collaborated in the study design. All authors reviewed the final manuscript.
